# Advancements and Frontiers in the High Performance of Natural Hydrogels for Cartilage Tissue Engineering

**DOI:** 10.3389/fchem.2020.00053

**Published:** 2020-02-12

**Authors:** Wuren Bao, Menglu Li, Yanyu Yang, Yi Wan, Xing Wang, Na Bi, Chunlin Li

**Affiliations:** ^1^School of Nursing, Inner Mongolia University for Nationalities, Tongliao, China; ^2^Beijing National Laboratory for Molecular Sciences, State Key Laboratory of Polymer Physics & Chemistry, Institute of Chemistry, Chinese Academy of Sciences, Beijing, China; ^3^College of Science and Engineering, Zhengzhou University, Zhengzhou, China; ^4^Orthopaedic Department, The 8th Medical Center of Chinese PLA General Hospital, Beijing, China; ^5^University of Chinese Academy of Sciences, Beijing, China

**Keywords:** natural hydrogel, mechanical property, hydrogel scaffolds, cartilage tissue engineering, regenerative medicine

## Abstract

Cartilage injury originating from trauma or osteoarthritis is a common joint disease that can bring about an increasing social and economic burden in modern society. On account of its avascular, neural, and lymphatic characteristics, the poor migration ability of chondrocytes, and a low number of progenitor cells, the self-healing ability of cartilage defects has been significantly limited. Natural hydrogels, occurring abundantly with characteristics such as high water absorption, biodegradation, adjustable porosity, and biocompatibility like that of the natural extracellular matrix (ECM), have been developed into one of the most suitable scaffold biomaterials for the regeneration of cartilage in material science and tissue engineering. Notably, natural hydrogels derived from sources such as animal or human cadaver tissues possess the bionic mechanical behaviors of physiological cartilage that are required for usage as articular cartilage substitutes, by which the enhanced chondrogenic phenotype ability may be achieved by facilely embedding living cells, controlling degradation profiles, and releasing stimulatory growth factors. Hence, we summarize an overview of strategies and developments of the various kinds and functions of natural hydrogels for cartilage tissue engineering in this review. The main concepts and recent essential research found that great challenges like vascularity, clinically relevant size, and mechanical performances were still difficult to overcome because the current limitations of technologies need to be severely addressed in practical settings, particularly in unpredictable preclinical trials and during future forays into cartilage regeneration using natural hydrogel scaffolds with high mechanical properties. Therefore, the grand aim of this current review is to underpin the importance of preparation, modification, and application for the high performance of natural hydrogels for cartilage tissue engineering, which has been achieved by presenting a promising avenue in various fields and postulating real-world respective potentials.

## Introduction

**Figure 1:**
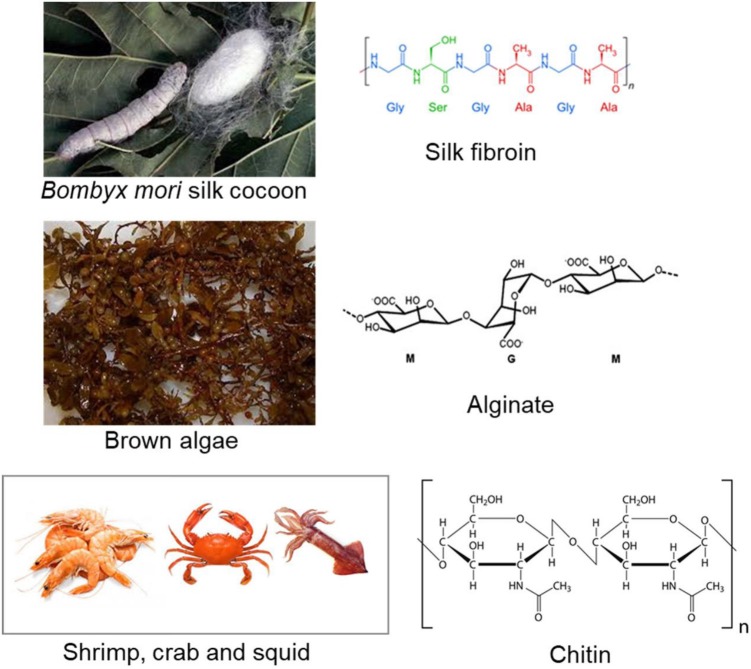
Some natural biopolymers, derived from renewable resources, and their respective chemical structures: silk fibroin, alginate, and chitin. Reproduced from Mano et al. (2007) with permission from Copyright 2007 Royal Society.

Natural polymeric materials are widely used in engineering and regenerating tissues for human health (e.g., skin, cartilage, bone, tracheal splints, and wound-healing vascular grafts) because of their unique advantages, namely biocompatiblity, biodegradation, favorable porsity, and achievable mechanics (Seal et al., 2001; Shin et al., 2003; Shelke et al., 2014; Sahana and Rekha, 2018; Zhang et al., 2019). Inspired by biological macromolecules within the extracellular matrix, natural polymers or biopolymers are generally obtained from various renewable resources, such as animals, plants, algae, and microorganisms found throughout the world (Figure 1), which can elude chronic inflammation toxicity or immunological reactions after suitable synthetic modification methodologies are applied. Thus, it is deemed that natural polymers are essential for designing bioactive compounds, for drug delivery systems for disease treatment, and for the construction of smart therapeutic systems for bioengineered functional tissues. In this case, the emergence of natural hydrogels (e.g., amino acids, proteins, polysaccharides, and glycosaminoglycans) has brought about significant clinical application values byimplant fabrication methods (Mano et al., 2007).

Regeneration of cartilage defects has historically been an enormous challenge to both orthopedic surgeons and patients,—it is reported that 60% of knee arthroscopy patients have cartilage injuries, wherein 15% of people (over 60 years old) have serious clinical features of cartilage injury (Hjelle et al., 2002; Cancedda et al., 2003; Ren et al., 2015; Walker and Madihally, 2015). Unlike most other tissues, cartilage is essentially avascular and low in cell content. Therefore, the lack of vascularization, innervation, lymphoid networks, and proper progenitor cells can greatly limit the ability of damaged cartilage to heal itself (Huey et al., 2012; Liao et al., 2014; Yuan et al., 2014; Vilela et al., 2015). Current strategies on cartilage tissue regeneration have exhibited great effects in clinical practice, including traditional microfracture (bone marrow stimulation) (Dorotka et al., 2005; Mithoefer et al., 2009), autologous chondrocyte implantation (Ruano-Ravina and Jato Diaz, 2006; Niemeyer et al., 2008; Selmi et al., 2008; Harris et al., 2010; Peterson et al., 2010), autologous osteochondral transplantation, and allogeneic osteochondral transplantation (Glenn et al., 2006; Benazzo et al., 2008; Haene et al., 2012), etc. However, there are still obvious limitations and deficiencies that include tedious *ex vivo* cell manipulation, potential tumorigenesis, therapeutic translation risk and regulatory approval. So, it is of great clinical significance to develop and achieve a method of complete and permanent repair of damaged cartilage.

Fortunately, tissue engineering, consisting of scaffolds, cells, and favorable growth factors, has evolved into a most promising therapeutic strategy for cartilage tissue reconstruction (Khan and Malik, 2012; Kim et al., 2012; Grottkau and Lin, 2013; Sahni et al., 2015; Wang et al., 2016). To achieve the perfectible regeneration of damaged cartilage, it is essential to offer the biodegradable scaffolds, simulate local characteristics of specific tissues, transport the tissue cells and growth factors, and provide supports to newly formed tissues (Malda et al., 2013). Ideally, cartilage tissue-engineered scaffolds should be porous, nontoxic, biocompatible, and biodegradable, and they should enhance cell differentiation and tissue generation, which need to possess high performance, matched rate between the degradation and new-tissue formation, diffused nutrients and metabolites behaviors, adhesion to the surrounding native tissue fusion, and fulfillment of the damaged sites (Hollister, 2005; Balakrishnan and Banerjee, 2011). For the construction of an ideal tissue engineering program, it is important to provide the functional biomaterials that basically mimic the natural ECM of cartilage components. Traditional approaches generally include direct implantation into tissue defects, precise incorporation of bioactive growth factor into the targeted tissues, cell-free scaffold biomaterials, and mimicking natural ECM with cell-laden architectural scaffolds, among which three-dimensional (3D) porous hydrogel scaffolds are most frequently used to promote cell organization into the extracellular matrix during reconstructive periods (Hubbell, 1995; Griffith and Naughton, 2002; Khademhosseini and Langer, 2006; Place et al., 2009; Berthiaume et al., 2011; O'brien, 2011).

Hydrogels, composed of natural or synthetic hydrophilic polymer strands connected with each other at crosslinking points, possess a unique 3D crosslinked polymeric network encompassing a wide range of chemical compositions and bulk physical properties. The hydrophilic nature of constituting polymeric chains allows the hydrogels to absorb amounts of water (more than 1,000-fold compared to their dry weight) to be applied in a variety of technological biomaterials for drug delivery and tissue regeneration, among which the *in situ* hydrogels possess the advantage of simple drug formulation and the ability to deliver both hydrophilic and hydrophobic drugs. Based on the cross-linking properties, hydrogels are classified into “chemical” and “physical” network gels. Chemically crosslinked hydrogels are generally held together by molecular bonds of synthetic polymers and possess stable, homogeneous, and adjustable structures. While physically crosslinked hydrogels are generally aggregated by secondary interactions such as molecular entangling, hydrogen bonds, ionic bonds, or hydrophobic interactions force them to form a reversible structure and self-healing properties, mainly including biodegradable natural polymers, which has several advantages over chemically cross-linked hydrogels, including solvent casting, easy fabrication, less toxic, reshaping, postprocess bulk modification, biodegradation, and so on (Eslahi et al., 2016; Li et al., 2019).

As a typical biological scaffold, hydrogels possess unique architectures of highly hydrated 3D and versatile capacities of high water content, suitable pore size and porosity, substance exchange capacity, good biodegradability performance, and extraordinary mechanical properties (Peppas et al., 2006), and can provide a suitable microenvironment and efficient biocompatibility and high strength for holding considerable promise in cartilage differentiation and cartilage-specific ECM regeneration, thus resulting in their wide usages for tissue engineering and cell therapy in various bio-applications. It is mentioned that the network pore of hydrogels played important roles in the physicochemical and mechanical signals and nutritive delivery for the cell growth. For example, pore size and high porosity were beneficial to the cell infiltration and ECM formation, while the interconnected and open pores could promote cell growth, proliferation, and migration, as well as the tissue vascularization process (Furth et al., 2007; Ma, 2008; Xiao et al., 2015). The micro-porosity was another important factor to facilitate the cell adhesion and spreading to improve the biomechanics between the hydrogel scaffolds and tissues (Karageorgiou and Kaplan, 2005; Loh and Choong, 2013). In addition, the composition, structure, biocompatibility, safety, stability, and mechanical properties of hydrogel scaffolds can also be considered to meet the needs of the cell morphology, proliferation, and differentiation in cartilage tissue regeneration for clinical scenarios (Wang et al., 2008, 2018; Spiller et al., 2011; Amini and Nair, 2012; Ji et al., 2012). As typical representatives, natural hydrogels with high performance are ideal biomaterial scaffolds for cartilage repair by their preferable reconstructions of cell growth, proliferation, and differentiation and new tissue formation.

This review will classify the preparation materials of hydrogels and summarize their typical kinds and wide applications of several typical natural hydrogels (alginate, chitosan, gelatin, collagen, hyaluronan, and natural hybrids) with good biocompatibility, improved stability, and high performance for facilitating cell delivery in the cartilage tissue engineering and regeneration medicine fields. We also summarize the different advantages, disadvantages, modification methods, and the future prospects of natural hydrogels for cartilage tissue engineering. Finally, we provide some suggestions and prospects on developing natural hydrogels via their tailored physicochemical and mechanical properties for effective cartilage tissue engineering. Understanding medical needs and concurrently lessening the difficulty of hydrogel construction should therefore be the goal for future research in this field.

## Classification of Hydrogels

Hydrogels can be briefly classified into synthetic and natural polymers for cartilage tissue regeneration in biomedical applications.

### Synthetic Polymers

Synthetic polymers have excellent characteristics in terms of molecular weight, degradation, and mechanical properties, with the advantage of having tailored property profiles for specific applications, exhibiting wide usage due to their controllability, reproducibility, and good mechanical properties. Representative synthetic polymers for tissue regeneration include polylactide (PLA), poly-lactide-co-glycolide (PLGA), polyglycolide (PGA), poly-(D,L-lactic acid) (PDLLA), polycaprolactone (PCL), poly-ethylene-glycol (PEG), poly(vinyl alcohol) (PVA), poly (N-isopropylacrylamide) (PNIPAM), and polyacrylamide (PAM). These polymers can be self-reinforced to enhance their mechanical strength. However, many of these polymers present an immune response or toxicity, particularly when combined with certain polymers and are not capable of being incorporated with host tissues. They exhibit lower biological activity because of their potential for a local pH increase by acidic degradation products, inflammatory response, poor degradation, and inflammation associated with high molecular weight polymers (Katti et al., 2002; Gunja and Athanasiou, 2006; Pina and Ferreira, 2012; Pereira et al., 2014).

### Natural Polymers

Natural polymers have explicit biomedical applications in tissue regeneration due to their biocompatibility, biodegradability, and macromolecular similarity to the original ECMs, which can provide a magnificent bioactivity and natural adhesive surface for cells required for bioactivity. Natural polymers used for hydrogel preparation include protein-based materials (such as gelatin, collagen, fibrin, and silk fibroin) and polysaccharide-based materials (such as hyaluronic acid, chondroitin sulfate, alginate, chitosan, and so on). In addition, natural hydrogels cannot cause immune and toxic reactions, and the degradation products are non-toxic and non-immunogenic, leading to the excretion of final metabolites outside the body safely; but, their poor stability, rapid degradation, and relatively low mechanical strength greatly limits their applications (Malafaya et al., 2007; Mano et al., 2007; Nair and Laurencin, 2007).

Although hybridization of synthetic and natural materials is an efficient and easygoing approach to integration, the advantages of constructing the hydrogels for the cartilage tissue regeneration, the undegradable components, and the unpredictable metabolites have still brought about significant limitation in actual biomedicine. Therefore, hybridization of other natural polymers or advanced biomodification of natural hydrogels to acquire better mechanics are the most promising strategies for constructing ideal biomaterial scaffolds to satisfy the requirements of cartilage repair by the designable and preferable reconstructions of cell morphology, growth, proliferation, differentiation, and new tissue formation.

## Natural Hydrogels for Cartilage Tissue Engineering

### Alginate

Alginate (ALG), as a natural polysaccharide extracted from brown algae, consists of 1,4-chain D-mannitol acid and L-gulu acid residues and has been widely applied to encapsulate the cells due to its good biocompatibility, high hydration viscoelasticity, and physically crosslinked ability (Pelletier et al., 2001; Hashimoto et al., 2004; Cho et al., 2005, 2009; Tritz et al., 2010; Zeng et al., 2014). Compared to other natural polymers, alginate is favorable for cell function and cell-immobilized microspheres or 3D porous hydrogel scaffolds (El Khoury et al., 2014; Zehnder et al., 2015). Alginate hydrogel can support the growth and proliferation of enveloped chondrocytes and maintain their chondrocyte morphology. For example, Swieszkowski et al. found that about 80% of human chondrocytes were retained in the 3D-deposited hydrogel filaments at 14 days after culture *in vitro*. Meanwhile, the embedded chondrocytes remained round in the whole culture processes (Kosik-Koziol et al., 2017). In addition, alginate hydrogels are also used to transport mesenchymal stem cells (MSCs) for cartilage regeneration. Wang et al. prepared a multiphasic graft by linkage of a cartilaginous alginate hydrogel and a sintered poly(lactic-co-glycolic acid) microsphere scaffold using a fibrotic cartilaginous ECM. Within this condition, these culturing chondrocytes could achieve the favorable gradient transition and integration from the cartilage layers to the subchondral bone layers, exhibiting the excellent tissue repair efficacy using a defected rabbit knee model (Fonseca et al., 2014).

**Figure 2:**
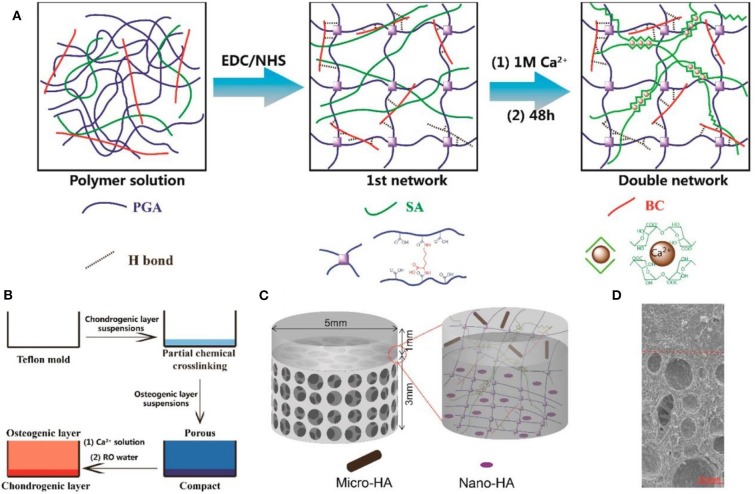
Schematic illustration of **(A)** preparation process of BC-DN hydrogels, bilayered hydrogel scaffolds, and the structure of bilayer hydrogel. **(B)** Schematic depiction of the preparation of bilayer hydrogel scaffolds. **(C)** Schematic illustration of the structure of the bilayer hydrogel. **(D)** SEM image of bilayer hydrogel scaffolds. Reproduced from Zhu X. B. et al. (2018) with permission from Copyright 2018 American Chemical Society.

However, there are still some limitations in tissue engineering applications. Firstly, the physically crosslinked alginate hydrogel possesses poor stability and gradually loses its mechanical strength within a short period of time, even in the physiological environments, which always require the subsequent crosslinking processes to strengthen mechanical property (Vallee et al., 2009). Secondly, on account of low cell adhesion and cell interaction ability of alginate in mammals, cell adhesion peptide is often introduced to better support cell function (Alsberg et al., 2001). In order to overcome these defects, other bioactive substances are usually added into alginate hydrogels. Sodium citrate was added into ALG as the dispersant of hydroxyapatite (HAP). Eames et al. found that the ALG/HAP complex could trigger the chondrocytes to secrete a calcified matrix, which was testified by the favorable survival and proliferation of chondrocytes in the ALG/HAP structure and high expression level of calcified cartilage markers (You et al., 2019). Embedding bone marrow-derived mesenchymal stem cells (bMSCs) in RGD (arginine/glycine/aspartic acid)-functionalized, γ-ray alginate hydrogels could enhance the osteochondral regeneration and promote the development of a more mechanically functional repair tissue (Critchley et al., 2019). However, the poor mechanical properties of ALG-based hydrogels limited their biomedical potential in osteochondral tissue regeneration. Lu et al. aimed to prepare a high-performance biohydrogel through introducing the bacterial cellulose (BC) into a double-network hydrogel system (Figure 2). The compressive modulus was matched with the natural articular cartilage, while their swelling degrees obviously declined. Then, a bilayer hydrogel scaffold was fabricated via chemical and physical crosslinking methods for achieving osteochondral regeneration on the basis of the bionics principle. After the addition of another two hydroxyapatite particles with varied sizes, the results of osteochondral defect model of rabbits verified the good osteochondral repair effects of these bilayer structural scaffolds (Zhu X. B. et al., 2018).

In addition to improving the mechanical properties and cell adhesion of alginate hydrogels, alginate hydrogels were prepared as carriers for encapsulating a variety of low molecular weight drugs. Partially oxidized alginate hydrogel could realize the drug control and local administration (Bouhadir et al., 2001; Colinet et al., 2009). Using the alginate-polymethacrylate hybrid hydrogels as the framework, the scaffold materials were prepared by crosslinking into a single porous structure on the basis of the electrostatic and covalent interactions, which overcame the mechanical property limitations of the pure alginate materials. Meanwhile, the alginate portion provided an appropriate microenvironment mimicking extracellular matrix, while the methacrylate portion could also improve the mechanical properties of resulting mixed hydrogels (Stagnaro et al., 2018).

### Chitosan

Chitosan is a kind of mucopolysaccharide widely existed in nature, which is important component of connective tissue with complexation, bacteriostasis, adsorption, and antioxidant effects (Molinaro et al., 2002; Jayakumar et al., 2005). Recent reports demonstrated that chitosan can be gelated in an acidic pH or a non-solvent condition (Ribeiro et al., 2017; Xu et al., 2017; Chen Y. R. et al., 2019), and further be prepared for chitosan-based hydrogel scaffolds. Chitosan has good biocompatibility and biodegradability; therefore, it is a kind of tissue-engineering material with wide application prospects and can be considered as a potential material for cartilage repair in regenerative medicine fields. A previous study demonstrated that since chitosan is extracted from shrimp shells, participants selected for this study were allergic to at least one type of shellfish or shrimp in order to test to see if they were allergic to chitosan. The results showed no adverse reactions among participants, providing the first evidence of biosafety of chitosan in allergic patients (Waibel et al., 2011). Although a simple mixture of chitosan with other natural polymers could generate a series of functional hydrogels via the electrostatic interactions (Ma et al., 2009), the physically crosslinked networks presented the terrible dissolution behaviors and weak mechanics that greatly limited their wide applications for artificial cartilage regeneration (Yang Y. Y. et al., 2018).

**Figure 3:**
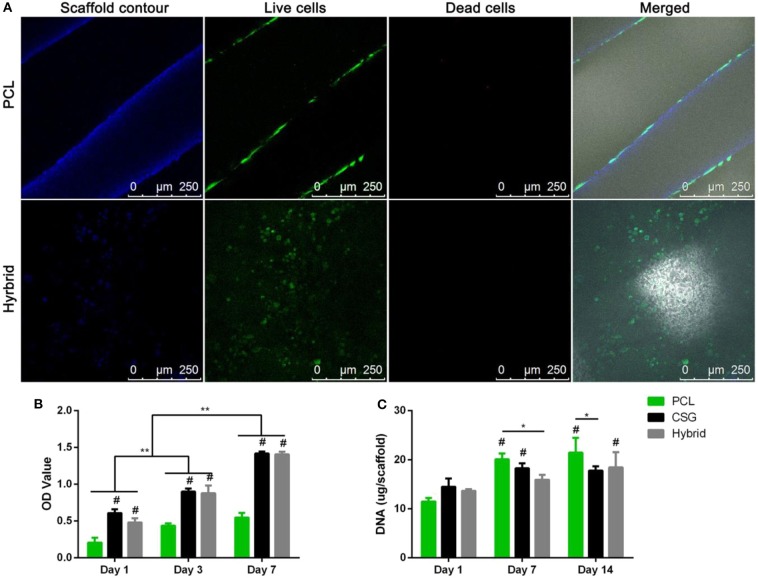
**(A)** Representative images of BMMSCs attachment, viability, and distribution in composite scaffolds. Blue fluorescence represents the contours of scaffolds; merged images include bright field views to show the scaffold pores. CLSM images of Live/Dead staining demonstrated cell viability of after 72 h of culture in growth medium. (Red represents the dead cells; green represents the live cells; Scale bar = 250 μm). CCK-8 assay showed that the number of cells in the three groups increased over time **(B)**. DNA content in the various scaffolds during osteogenic culture indicating slow proliferation while MSCs differentiating into osteoblasts. Results are expressed as mean ± SD (*n* = 3, ^*,#^*P* < 0.05, ^**,*##*^*P* < 0.01; ^#^compared to PCL group in **(B)**, and compared to day 1 in **(C)**). Reproduced from Dong et al. (2017) with permission from Copyright 2017 Springer Nature.

To overcome the flaw of water insolubility, N-succinyl chitosan-dialdehyde starch mixture hydrogel was prepared with good solubility to repair cartilage defects (Kamoun, 2016). In addition, the sensitization and mechanism of chitosan may also need improvement in its clinical transformation. Yu et al. found that when the thermo-responsive chitosan-based hydrogels are introduced into the 3D-printed PCL scaffolds to form the composite scaffolds, the composite scaffold has good cell- and drug-carrying capacity and good mechanical strength (Dong et al., 2017). Compared with pure chitosan hydrogels, the compressive modulus of the hybrid scaffold increased significantly after the introduction of PCL scaffolds (Figure 3A). These hybrid scaffolds are beneficial to cell survival. After culturing in growth medium for 72 h, BMMSCs survived in both PCL and hybrid scaffolds with a lot of dead cells, but their distribution patterns were different. Importantly, it was found that those encapsulated cells in hybrid scaffolds could not only distribute evenly in the pores but also spread on the surfaces of PCL scaffolds. A CCK assay provided consistent results indicating that these cell-scaffold composites *in vitro* culture exhibited the active proliferation for as long as 7 days (Figure 3B). Compared to the PCL scaffold, the cell number was greater in hydrogel and hydrogel-filled scaffolds at every time point, which indicated the excellent biocompatibility of hybrid hydrogel scaffolds compared to the highly hydrated environment of the single hydrophobic PCL scaffold. Therefore, these hybrid hydrogel scaffolds that have the satisfactory mechanical strength exhibited the favorable biomimetic micro-environments to facilitate the cell retention, growth, and distribution (Figure 3C).

Lee et al. prepared a smart biofunctional hydrogel for cartilage regeneration by photopolymerization of chitosan (MeGC) solution with Col II solution. TGF-β1 was conjugated into MeGC via SMCC moiety. The hydrogel system had no effect on the viability of loaded synovial mesenchymal stem cells. Compared with the pure chitosan hydrogels, the aggregation and deposition of mesenchymal stem cells were enhanced (Kim et al., 2015). Allogeneic chondrocytes were transplanted with chitosan-demineralized bone matrix composite hydrogel scaffold for cartilage injury therapy in rabbits. At 24 weeks after surgery, the cartilage defect was successfully filled and no obvious inflammatory reaction was observed (Man et al., 2016).

### Gelatin

**Figure 4:**
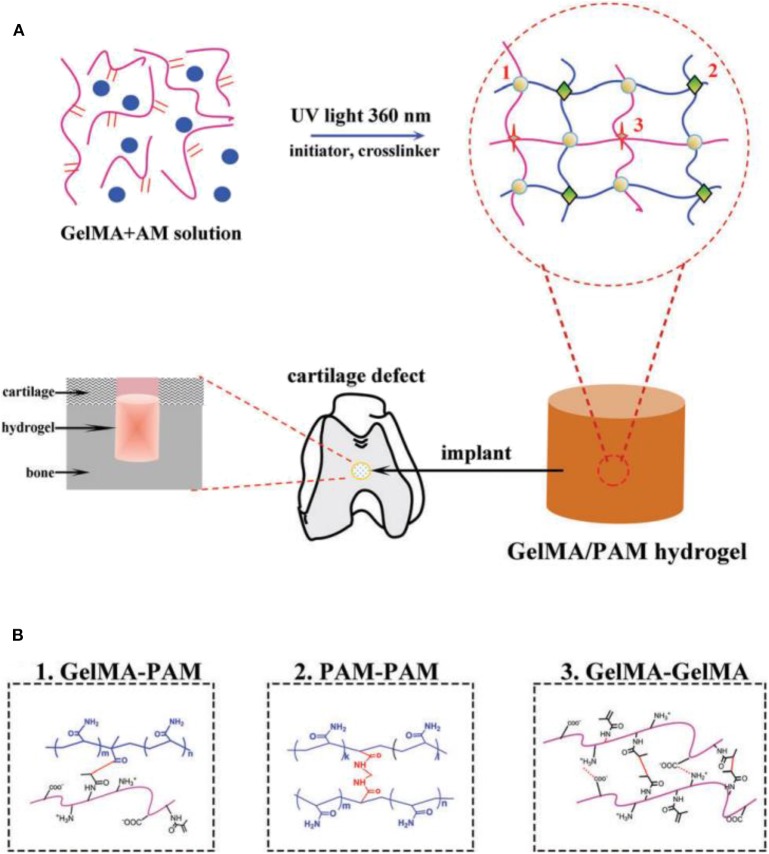
**(A)** Schematic illustration for fabricating natural-synthetic GelMA-PAM biohybrid hydrogel via the photo-initiating polymerization. **(B)** Molecular crosslinking structures: covalent crosslinking between GelMA-PAM, covalent crosslinking between PAM-PAM, and covalent/physical crosslinking between GelMA-GelMA. Reproduced from Han et al. (2017) with permission from Copyright 2017 Royal Society of Chemistry.

Gelatin is composed of a series of arginine-glycine-aspartic acid sequences that can benefit to improve the cell adhesion and matrix metalloproteinases capacities. Thermal reversible changes occurred in gelatin solution at 30–40°C, and crosslinked hydrogels can be physically formed by the self-gelation effects or chemically generated by the chemical reactions (Sakai et al., 2009; Liu et al., 2016; Zhu et al., 2019). Up to now, the gelatin-based composites have been utilized as suitable scaffolds for tissue engineering and molecule carriers in biomaterial fields. Gelatin-based hydrogels have good biodegradability, biocompatibility, and cell/tissue affinity, but poor mechanical strength and low thermal stability greatly limited their applications in biomedical cartilage repair. Fortunately, a star product of gelatin methacrylamide (GelMA) was prepared by modifying gelatin with methacrylate anhydride that exhibited the significant roles in the cartilage tissue engineering applications. Under the action of a photo-initiator, acrylamide (AM) was copolymerized with gel under ultraviolet radiation to prepare gel-based natural synthetic polymer biohybrid hydrogel. These hybrid hydrogels had better mechanical properties, degradation rate, cell adhesion, and biocompatibility (Figure 4; Han et al., 2017). A new cell-laden cartilage structure was prepared by a tabletop stereolithography-based 3D bioprinter, which was composed of methacrylate, polyethylene glycol diacrylate biocompatible photo-initiator, and transformed growth factor-1 embedded nanospheres. The cell vitality and proliferation rate can be tailored by regulating the component content for the promising cartilage regeneration (Zhu W. et al., 2018). Furthermore, the chemical modification of gelatin combined with 3D printing technology to prepare biological scaffolds provided a new idea for the treatment of osteoarthritis. Some researchers also had designed biological scaffolds for the transport of extracellular matrix extraneous bodies like ECM/GelMA/exosome scaffolds (Chen P. F. et al., 2019).

**Figure 5:**
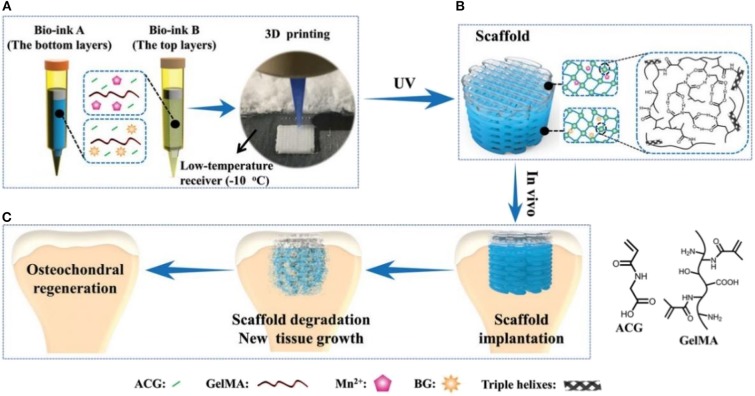
Schematic illustration of the biohybrid gradient scaffolds for osteochondral repair by 3D printing technology. **(A)** The compositions of bio-ink A and bio-ink B and 3D-printing method of hybrid gradient scaffolds assisted with a low-temperature receiver. **(B)** Formation of hydrogel scaffold after UV light-initiated polymerization with hydrogen bonding interactions. **(C)** Osteochondral repair treated with the biohybrid gradient PACG-GelMA scaffold, with Mn^2+^ and BG being loaded on the top and bottom layers, respectively. Reproduced from Gao et al. (2019) with permission from Copyright 2019 Wiley.

Although GelMA hydrogels exhibited obvious advantages in tissue engineering, they were still lacking in high mechanical properties for achieving the efficient cartilage regeneration only by the pure GelMA hydrogels. Therefore, Liu et al. constructed a biodegradable hydrogel via the photo-initiated polymerization of poly(N-acryloyl 2-glycine) (PACG) and GelMA (PACG-GelMA) (Figure 5), which possessed high mechanical strengths, with a tensile strength of 1.1 MPa, outstanding compressive strength of 12.4 MPa, large Young's modulus of 0.32 MPa, and high-compression modulus of 0.837 MPa. By tailoring the ACG/GelMA ratios, the temporary PACG network was stabilized by chemical crosslinking effects, thus exhibiting the adjusting biodegradability. Furthermore, they fabricated a biocompatible composite scaffold with PACG-GelMA hydrogel-bioactive glass and PACG-GelMA hydrogel-Mn^2+^ layers for osteochondral repair using 3D printing techniques. *In vitro* and *in vivo* biological results demonstrate that these biocompatible hybrid gradient hydrogel scaffolds could facilitate cell adhesion, spreading, osteogenic-oriented differentiation, gene expression, cartilage regeneration, and subchondral bone formation in a rat model (Gao et al., 2019).

Chemically modified gelatin-based hydrogel is biodegradable, and its properties are adjustable and easily micro-processed. However, further improvement is needed in forming cartilage and simulating the function of cartilage tissue (Yang et al., 2017). First, the activation of chondrocytes in the gel should be enhanced to obtain more connective tissue ECM. Second, their mechanics should be further optimized and improved. Third, it is necessary to facilely adjust the rheological properties of modified gelatin hydrogel prepolymer solutions. To achieve these goals, adding other functional components as needed will be an efficient method to enhance the properties of gelation-based hydrogels. Lu et al. developed a novel mussel-inspired strategy to improve the mechanics of GelMA hydrogels by incorporating dopamine methacrylate (ODMA) oligomers into the GelMA chains. Intercalation of ODMA made the GelMA hydrogels resilient and stable at body temperature by reducing the entangled GelMA chain density and introducing other sacrificial physical crosslinking interactions. *In vitro* and *in vivo* experiments verified that this modified ODMA-GelMA hydrogel, as a typical growth factor-free scaffold, not only provided a favorable microenvironment to promote the mesenchymal stem cell attachment and spreading, but also enhanced the cartilage regeneration after encapsulation of chondroitin sulfate or TGF-β3, which would be served as an ideal candidate hydrogel scaffold for cartilage or other tissues repair in biomedical applications (Gan et al., 2019).

### Collagen

Collagen, as an important component of extracellular matrix, is a natural biological material, widely found in skin, bone, cartilage, blood vessels, teeth, and tendons, which had been widely used in biological and medical fields. Collagen hydrogels could be prepared by the UV irradiation photopolymerization, dehydrogenation heat treatment or other crosslinking reactions with aldehydes, carbimines, genipin, isocyanates, transglutaminase, etc. (Zhao et al., 2013). Collagen type I hydrogels could support mesenchymal stem cell adhesion, growth, spreading, and cartilage differentiation for the construction of engineered osteochondral structures *in vitro* (Wang et al., 2019). Giuffrida et al. had assessed a new kind of 3D scaffold that consisted of type I collagen and human adipose-derived mesenchymal stem cells, which exhibited the favorable chondrogenic potentials. Regardless of the presence of chondrogenic inducing factors, the scaffold had a higher potential for cartilage regeneration (Calabrese et al., 2017). *In vivo* and *in vitro* experiments showed that type II collagen hydrogels containing chondrocytes supported the proliferation and chondrogenesis of mesenchymal stem cells (Pulkkinen et al., 2010; Ren et al., 2016). It was found that bovine mesenchymal stem cells were cultured in monolayer, alginate, and type II collagen hydrogel. Cell differentiation of type II collagen hydrogel was the most obvious, and the cell differentiation was time-dependent. These type II collagen hydrogels had the potential to maintain the cartilage formation in mesenchymal stem cells (Bosnakovski et al., 2006). Besides, the hybrid hydrogel prepared by type I and type II collagen could regulate the performance of the hybrid hydrogels by adjusting the content of two types of collagen. The results showed that the higher the compression modulus of hybrid hydrogel was, the more extracellular matrix the chondrocytes secreted (Yuan et al., 2016).

**Figure 6:**
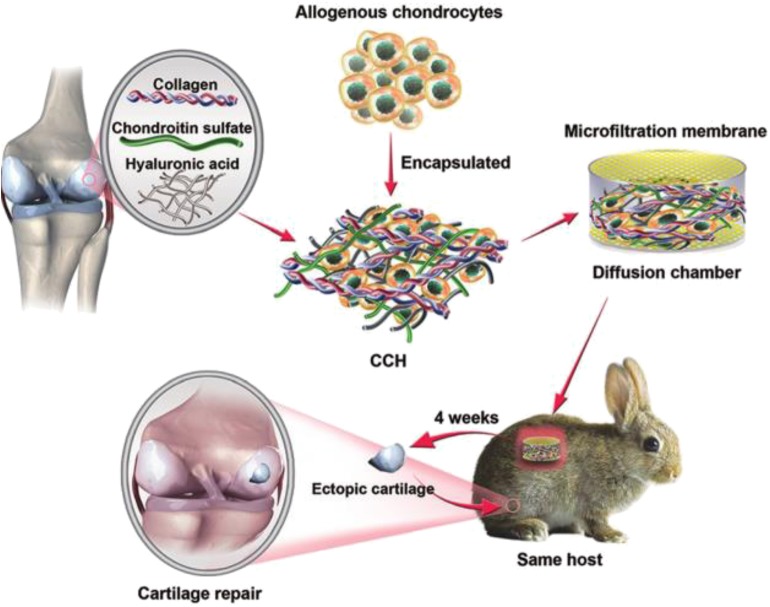
Schematic illustration of the overall design of three-phase hybrid hydrogels. Allogeneic chondrocytes are encapsulated with a CCH hybrid hydrogel, forming the ectopic cartilage with a diffusion chamber system for cartilage repair. Reproduced from Jiang et al. (2018) with permission from Copyright 2018 Royal Society of Chemistry.

It is common to combine collagen with other natural biological macromolecules to prepare hybrid hydrogels by the typical chemical modification of collagen. It was shown that the preparation of hyaluronic acid and collagen hybrid scaffold material with prednisone as anti-inflammatory drug was an ideal choice for cartilage regeneration in osteoarthritis and for the sustained release system of prednisone (Mohammadi et al., 2018). Type II collagen and hyaluronic acid could prepare the injectable hydrogels *in situ*, followed by the encapsulation of cartilage cells. Chondrocytes remained alive during culture and maintained the phenotypic characteristics of chondrocytes. In addition, the expression of the chondrocyte specific genes increased with time (Kontturi et al., 2014). Biological scaffolds were prepared by mixing type I collagen with sodium alginate as 3D bioprinting ink. The mechanical strength of the scaffold was improved, and it could significantly promote the cell adhesion/growth, improve the cell proliferation, and enhance the specific gene expression of cartilage (Yang X. C. et al., 2018). After encapsulation with allogeneic chondrocytes, three-phase synthetic collagens, chondroitin sulfate, and hyaluronic acid hydrogels (CCH) were transplanted into cartilage defects, demonstrating that hybrid collagen hydrogels exhibited higher cartilage specific markers of cell growth, proliferation, GAG secretion, and gene/protein expression, which was closer to natural cartilage matrix than collagen hydrogel (Figure 6; Jiang et al., 2018).

**Figure 7:**
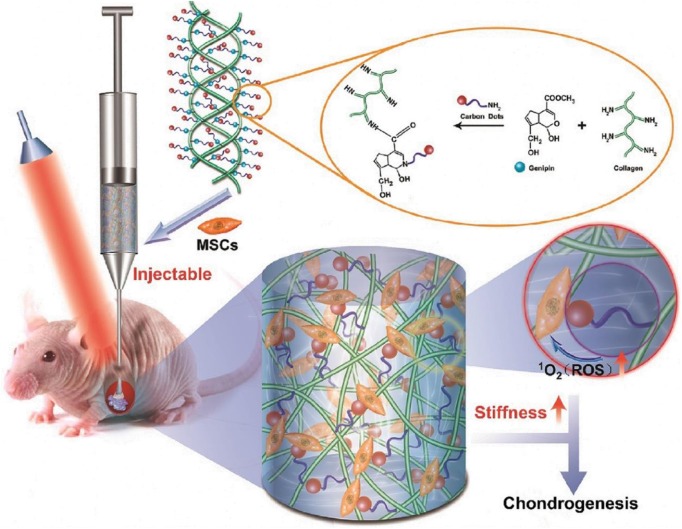
Schematic illustration of the fabrication process and implementation of CGN nanocomposite hydrogels. Reproduced from Lu et al. (2019) with permission from Copyright 2019 Elsevier.

Although collagen has been applied for the application of cartilage repair, its low stiffness and rapid degradation was not beneficial for chondrogenesis. Li et al. developed a kind of injectable collagen hydrogel of collagen-genipin-CD nanoparticles (CGN) through crosslinking the carbon dot nanoparticles (CD NPs) onto collagen with biocompatible crosslinker of genipin. On account of the effective linkage of genipin and CD NPs, these hydrogels showed high stiffness and produced a number of reactive oxygen species (ROS) by the photodynamic therapy (PDT). The organic combination of PDT and CGN hydrogel could obviously increase the BMSCs proliferation, upregulate the cartilage-specific gene expression, enhance the GAG secretion, and accelerate the cartilage regeneration within 8 weeks (Figure 7), which was attributed to the chondrogenic differentiation from the synergistic stiffness enhancement and ROS generation effect. So, this organic combination on the hydrogel injection and PDT treatment will represent a novel kind of strategy for the cartilage repair applications (Lu et al., 2019). In addition, enhancement of linkage interface between collagen hydrogels and bone-like substrates was also important for the regenerative medicine, because it is inevitable to use the heterogeneous scaffolds to achieve their multifunctionally gradient properties when the tissue cannot be completely repaired by a homogeneous graft. Therefore, improvement of the contact interface among the various layers is critical to construct the advanced hydrogel scaffolds with optimal performances. Borros et al. developed a pentafluorophenyl methacrylate (PFM) coating method through the immobilization of collagen-based hydrogels onto the desired substrate, because of high reactivity of PFM-coated substrate toward amines; in this case, the hybrid hydrogels were subsequently fibrillated and finally formed (Mas-Vinyals et al., 2019).

### Hyaluronan

Hyaluronate (HA) is a typically linear polysaccharide formed by 250–25,000 repeated disaccharide units consisting of N-acetylglucosamine and D-glucuronic acid, which is a crucial component of ECM and plays an important role in cell signal transduction and wound healing (Tool, 2001; Toole, 2004). Therefore, HA-based hydrogel is recognized as one of the most promising natural materials for cartilage tissue engineering. Owing to the unique effect of hyaluronic acid on the formation of chondrocytes, the application of HA-based hydrogel containing chondrocytes has been widely studied in cartilage tissue regeneration (Barbucci et al., 2002; Chung et al., 2006; Kang et al., 2009). Hyaluronic acid was chemically modified to form derivatives with better biocompatibility and controllable biodegradation. A biocompatible *in situ* crosslinked HA hydrogel can be obtained by the biological orthogonal reaction. The hydrogel is formed by a copper-free click-reaction between the azide and dibenzyl cyclooctane, which was proven to be an injectable scaffold *in vitro* and *in vivo* (Han et al., 2018). An *in situ* photo-crosslinked hyaluronic acid was developed as a scaffold material for articular cartilage repair. The physical and mechanical properties of these crosslinking hyaluronate hydrogels are similar to the other natural hydrogels. The chondrocytes were embedded in hydrogels and cultured *in vitro*. The cells remained round and accumulated a large amount of cartilage matrix. The hydrogel was inserted into the cartilage defect, and a large amount of cartilage matrix accumulated within 2 weeks after the surgery (Nettles et al., 2004). In the other method, HA was modified by the methacrylate anhydride and photopolymerized into a network with extensive physical properties. The volume expansion rate of the network was distributed between 8 and 42%, the compression modulus was 2–100 kPa, and the degradation time increased from <1 d to nearly 38 days (Burdick et al., 2005). Later, some studies showed that using visible green light instead of ultraviolet light to activate the crosslinked system would not damage the properties of materials. The compression modulus of hydrogel network can be adjusted to 3–146 kPa (Fenn and Oldinski, 2016). Therefore, the hyaluronic acid hydrogels prepared by chemical modification have adjustable biodegradability and mechanical properties and better optical crosslinking ability.

**Figure 8:**
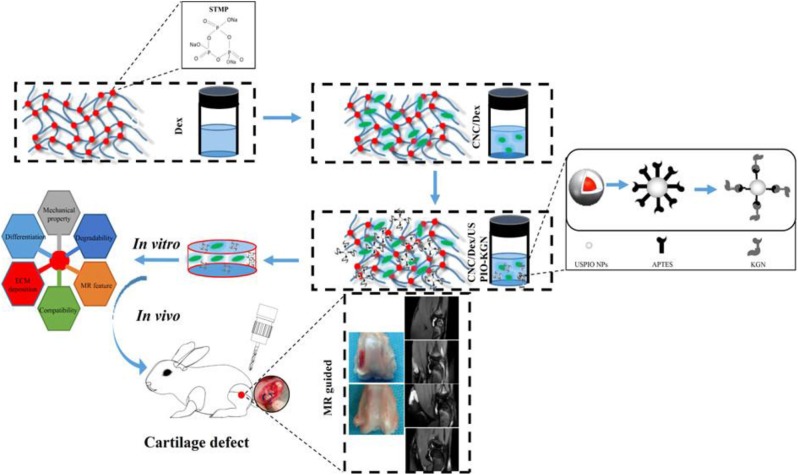
Schematic illustration of preparation and utilization of USPIO-labeled Dex/CNC/USPIO-KGN hydrogels for artificial cartilage repair. Reproduced from Yang et al. (2019) with permission from Copyright 2019 American Chemical Society.

In terms of the biological safety of implantation materials, the extracellular matrix degradability has gained increasing attention in tissue engineering. Cheng et al. prepared a completely bio degradable hydrogel by combining synthetic and natural polysaccharide polymers with their respective features. By mixing polyphosphate copolymer poly(butynyl phospholane)-random-poly(ethylethylene phosphate) (PBYP-r-PEEP) with thiolated hyaluronic acid (HA-SH) via the thiolyne “click” reaction, the fabricated HA/PPE hydrogel, supporting the human mesenchymal stem cells (hMSCs) adhesion and growth, could promote the cell-cell interactions with the enzymatic biodegradability and expand the range of biodegradable biomaterials for tissue engineering (Hao et al., 2019). In addition, Guo et al. prepared a kind of USPIO-KGN for cartilage repair by means of a stable non-protein compound of kartogenin (KGN) that promoted the BMSCs differentiation into chondrocytes via the grafting onto surface of ultrasmall superparamagnetic iron-oxide (USPIO) to finally integrate into the cellulose nanocrystal/dextran hydrogels (Figure 8). It was found that KGN was sustainably released for a long time, thus recruiting endogenous host cells and inducing the BMSCs differentiation into the chondrocytes for achieving the effective cartilage regeneration, with verification of both *in vitro* and *in vivo* experiments (Yang et al., 2019).

### Natural Hybrid Hydrogels

**Figure 9:**
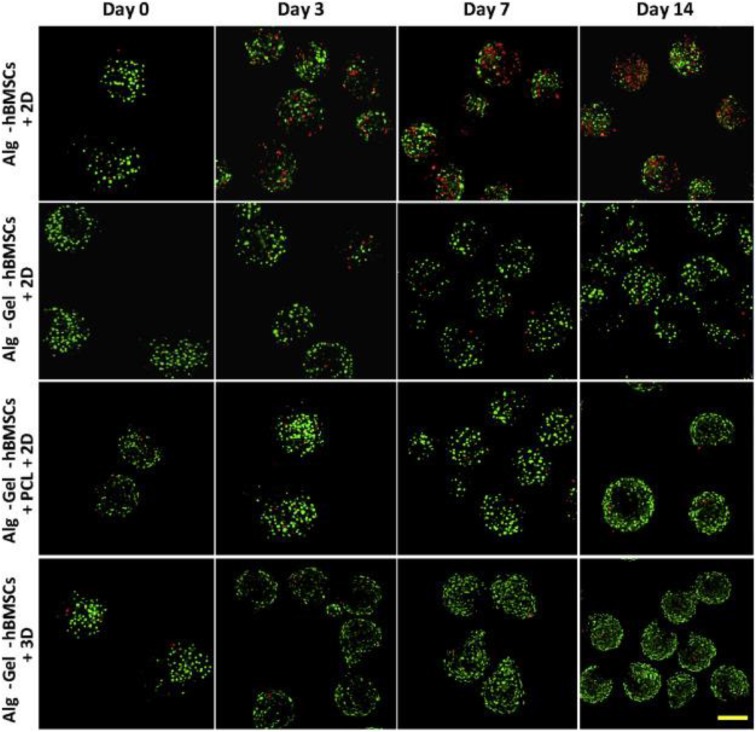
Live/Dead staining of ALG-hBMSCs, ALG-Gel-hBMSCs, and ALG-Gel-hBMSCs/3D-printed PCL scaffold with 2D well plate culture and ALG-Gel-hBMSCs with 3D bioreactor culture on day 0, 3, 7, and 14, respectively. Scale bar = 200 μm. Reproduced from Xu et al. (2019) with permission from Copyright 2019 Elsevier.

**Figure 10:**
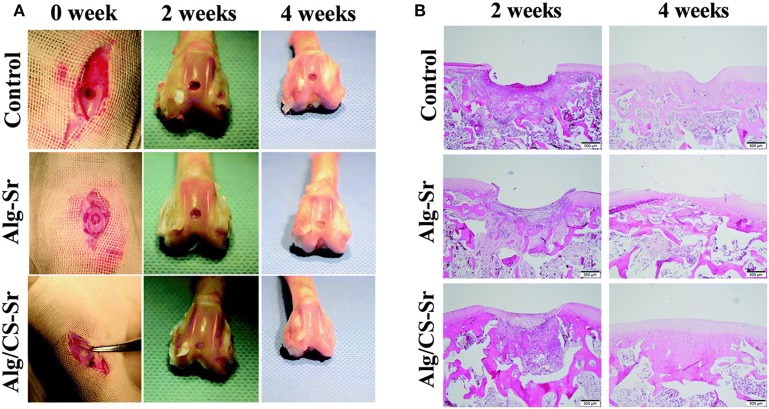
ALG hydrogel aiding cartilage defect repair in a rabbit model: **(A)** photographs of knee joints in the control group and the ALG hydrogel group at the 0, 2, and 4 weeks after post-operation. **(B)** H&E staining of cartilage defect. Reproduced from Ma et al. (2019) with permission from Copyright 2019 Royal Society of Chemistry.

Combination of various natural hydrogels is an ideal strategy for fabrication of smart and excellent hybrid scaffolds with high performance for cartilage tissue engineering and biomaterial fields. Lee et al. demonstrated a covalent method for conjugation of ALG to HA, to form the hyaluronate-alginate hybrid hydrogel (HAH) with the crosslinker of ethylenediamine in the Ca^2+^ solutions, which exhibited great potential as a scaffold for cartilage regeneration. Also, they used various types of linkers to obtain another series of HAH hydrogels by physical crosslinking methods. The mechanical property of HAH hydrogel was feasibly tailored by manipulating the linker between ALG and HA. Meanwhile, various linkers within the HAHs cultured in HAH hydrogel could also affect the chondrogenic differentiation of ATDC5 cells and be employed to fabricate the multifunctional scaffolds for cartilage regeneration (Park and Lee, 2014). Eglin et al. reported an optimization of human bone marrow stromal cell (hBMSC)-loaded alginate-gelatin microspheres within the 3D-printed PCL scaffolds for construction of mechanically stabilized and biologically supportive tissue engineering of cartilage (Figure 9; Xu et al., 2019). Tunable mechanical properties of composite hydrogels are important for biomedical applications. Tang et al. synthesized the strontium alginate/chondroitin sulfate (ALG/CS-Sr) composite hydrogels and analyzed the effect of strontium chloride concentration on the dynamically mechanical property. Cell viability assay revealed the good cytocompatibility of this hydrogel with the adequate characterization of flow cytometry, qPCR, and western blotting analysis, which verified this kind of composite ALG/CS-Sr hydrogel could exert a positive effect on the apoptosis inhibition with the anti-inflammatory effects in articular cartilage regeneration fields (Figure 10; Ma et al., 2019).

## Mechanics of Natural Hydrogels for Cartilage Tissue Engineering

Conventional hydrogels normally possess breakable characters that will decrease their stability and thus cannot be utilized for specific tissue applications such as bone, cartilage, and tendon. To overcome this issue, two effective strategies have been developed for cartilage tissue engineering. One is the hybridization of hydrogels with other polymers, nanoparticles, or nanofibers. For example, regenerated silk fibroin and chitin nanofiber have been used to improve the mechanical strength of GelMA hydrogels by β-sheet folding and self-assembly, respectively. The hydrogel elastic modulus increases by 1,000-fold, and strain-to-failure enhances by around 200% after chitin nanofiber assembly (Hassanzadeh et al., 2016). The hydrogels also demonstrate good cell viability, promotive cell differentiation, and stable vasculature formation. Collagen-based hydrogels with a 10-fold increase in stiffness have been realized after mixing very low amount of chemically functionalized nanoparticles as crosslinker epicenters to make collagen chains crosslinked on the surface of nanoparticles (Jaiswal et al., 2016). On account of the interactions between nanoparticles and polymer chains, the mechanical properties of hybrid hydrogels can be enhanced. The other strategy is to prepare interpenetrating polymer network (IPN) hydrogels with high mechanics and fracture strength, which has gained a lot of attention for cartilage tissue engineering (Dragan, 2014). Double networks (DN) are introduced in hydrogels to enhance mechanical property for cartilage tissue engineering (Gong et al., 2003; Yasuda et al., 2009; Fukui et al., 2014). The feature of DN hydrogels is the formulation of, first, a densely crosslinked hydrogel, and second, a loose network. The first network serves as sacrificial bonds to disperse the stress, while the second polymer chains work as hidden length that can extend to sustain large deformation (Haque et al., 2012). Similarly, ionic crosslinked chitosan with low molecular weight is used to work as the second crosslinking component to enhance the mechanical strength of the UV-initiated PAM hydrogel (Ma et al., 2009; Li et al., 2018).

However, due to the big gap of mechanical property between the ordinary hydrogel materials and human tissues, scientists have been seeking to improve mechanical strength of the hydrogels in recent years. Generally, there are several different ways that have been proven to enhance the mechanical strength of the hydrogels, including increasing the crosslink density, reducing the gel swelling degree, introducing the fibrous reinforcing agent and the preparation of interpenetrating networks (Anseth et al., 1996; Haraguchi and Takehisa, 2002; Sakai et al., 2008; Hunt et al., 2014; Ahadian et al., 2015; Hao et al., 2017). Especially, double network (DN) hydrogel provides an excellent idea to gain high strength for cartilage tissue engineering (Chen et al., 2015; Higa et al., 2016; Yan et al., 2017). DN hydrogel possesses two different types of network structures: the highly crosslinked polyelectrolyte networks and the lowly crosslinked or non-crosslinked neutral network structures. The former provides a rigid bracket for DN hydrogels, while the latter fills in rigid network and absorbs external stress (Sun et al., 2012; Yang et al., 2016; Golafshan et al., 2017). Until now, however, the study of the DN hydrogels in the field of biological materials, especially in the field of for cartilage tissue engineering applications are still at the initial stages with sums of challenges (Gu et al., 2018).

## Summary and Perspectives

This paper reviews the advancements of several mechanically natural hydrogel biomaterials designed and applied in cartilage tissue engineering in recent years. It has been found that the high performance of natural hydrogels has better biocompatibility and biodegradability and is more conducive to cell survival. One of the keys to cartilage tissue regeneration is to promote cartilage integration as well as subchondral bone regeneration, because these two tissues have various topological structures and moduli that requires the hydrogel scaffolds to simulate different structures and functions simultaneously. In addition, the cartilage repair effect is associated with the deposition and remodeling of ECM of the cartilage cells. If the degradation rate of repaired material is not well-matched, the ECM cannot deposit in the defect area that is harming the cartilage regeneration. Based on this feature, natural hydrogel has a controllable degradation rate, good biocompatibility, and outstanding mechanical property, so it is an ideal cartilage tissue engineering material. Meanwhile, high mechanism of hydrogel scaffolds loaded with regenerative drugs or cell that promote cartilage regeneration have been widely used in recent years. We only selected and highlighted some typical examples to raise the reader's interest and awareness about the high performance of natural hydrogels for cartilage tissue engineering.

With the development of tissue engineering and the regenerative medicine, it has been found that tissue regeneration and reconstruction require a multifunctional scaffold to load and deliver tissue-specific cells; in this case, hydrogel scaffolds are recognized as ideal biomaterials for tissue engineering like cartilage, bone, skin, heart valves, nerves, tendons, etc., because the composition, structure, morphology, function, and mechanics are closely similar to the natural tissue extracellular matrix. The natural hydrogels and 3D architecture scaffolds combined with various bioactive molecules, genes, and cells, as well as the tunable mechanical properties, have the capacity to guide and promote the *in vivo* implantation and development of multifunctional engineered tissues. Thus, these natural hydrogel scaffolds with customized morphologies and suitable mechanical behaviors are a series of exciting prospects in cartilage tissue engineering by tailorable retention and delivery abilities of cells and growth factors within the injury site, thus realizing the cell adhesion, growth, spreading, and differentiation, as well as the extensive applications, hereafter referred to as biohydrogels with high mechanical properties in tissue augmentation, repair, reconstruction, and regeneration.

Yet, it should be noted that it remains a major challenge to fully restore cartilage to its original composition, architecture, mechanics, and biofunction. For example, simultaneous achievement of integrating cartilage and subchondral bone regeneration has been a critical challenge in tissue engineering, because the difference in structure and modulus represents two distinct types of tissues that should be carefully considered to overcome the difficulties in simulating the structures and functions of the hybrid or bi-phase hydrogel scaffolds. In this, the part of cartilage repair exhibited a highly elastic modulus to bear pressure and resist friction and to facilitate the extracellular matrix, enhance the chondrogenesis expression of MSCs or chondrocytes, inhibit the hypertrophic differentiation, and contribute to the chondrocyte mineralization. Another part of subchondral bone repair could effectively contribute to the formation of a blood vessel network within the hydrogels to facilitate nutrient transport, stimulate osteoblast proliferations, and provide great support for regenerative cartilage. More importantly, integration of the surrounding cartilage and the implants should possess strong interfacial adhesion that can be significantly considered and enhanced for regenerated cartilage. Additionally, smart incorporations of intelligent or self-guided features like self-assembly and/or functional flexibility for dynamic biological demands also played essential roles in the fabrication and development of a new kind of high-performance natural hydrogel to obtain the full cartilage regeneration in biomedical applications.

It should be further noted that although there have been some limited clinically approved tissue-engineered products for the clinical trials status quo in recent years, a rapid progress toward more advanced and targeted therapies is still of note in promoting microfabrication techniques and developing the cellular scaffold-based approaches. It is concluded that an ideal natural hydrogel for achieving the cartilage tissue engineering should synchronously possess the following characterizations: (1) biological activity and biomimetic function; (2) mechanical reinforcement; (3) integration of cartilage with bone tissue, and (4) transport of drugs and growth factors. Therefore, the intelligent and hybrid hydrogel scaffolds with complex architectures should be well-fabricated for realizing the customized clinic treatments, and the corresponding research on the mechanical and biological behaviors of hydrogel scaffolds should also be emphasized to ensure powerful tissue interactions, resorption, and hierarchical architecture for enabling the tissue engineering implants. With this understanding, future work should forcefully focus on identifying the secondary, tertiary, and higher order architectures of hybrid nature-derived hydrogels, quantifying their composition, morphology and function, characterizing their binding pockets and interactions with cell surface receptors, and finally, turning them into a clinical tissue engineering biomaterial for effective cartilage tissue engineering. In this sense, we will establish such methodology or criteria on the design and development of final biological tissue engineering products for regenerative medicine, which makes natural hydrogel scaffolds more advantageous on adjustable structure, better strength, adequate immune response, adhesive interfacial binding force, and good biodegradability for enabling real applications in human patients.

This exciting goal will hopefully be achieved by the scientific community with the lessons learned from the literature in this review. Therefore, we are strongly convinced that with the help of continuous developments of natural hydrogels and exquisite adjustment of their physicochemical and mechanical properties for effective cartilage tissue engineering, more advanced multi-responsive histological engineering products with optimized architectures and functions will be eventually created to obtain greater manipulation and higher availability for various biomedical applications. While newly responsive hydrogel compositions, structures and mechanical properties will hopefully be continually developed, and the ability to obtain smart biomaterials with topological complexity is excitingly expanding in the next generation of outstanding tissue engineering products.

## Author Contributions

XW, NB, and CL conceived and designed the content of the paper. WB, ML, and YY collected the researched literatures, arranged the outline of collected documents, and wrote the articles. YW made important suggestions and helped revise the paper. All authors reviewed and commented on the entire manuscript.

### Conflict of Interest

The authors declare that the research was conducted in the absence of any commercial or financial relationships that could be construed as a potential conflict of interest.
